# Simple-minded solutions to complex problems

**DOI:** 10.3325/cmj.2022.63.399

**Published:** 2022-08

**Authors:** Charles H. Calisher

There are three major and many minor global problems today: the coronavirus pandemic and other global health threats, global climate change, and the Russian invasion of Ukraine being among them. The first is a long-term and continuing problem, the second is a devastating problem, and the last is an acute problem. There are others as well; there always are.

## CORONAVIRUS PANDEMIC

This is the major current problem. Although it has been reported by the World Health Organization that the global human cases total more than 530 million and world death toll from this disease probably has been upwards of 18 million people (1), the actual death toll may be more than 20 million, if one includes excess deaths largely attributable to overburdened health systems.

The picture in the USA alone is grim. We have passed 1 million deaths from COVID-19. It has killed one in 75 older Americans. Residents of long-term care facilities comprise less than 3% of the population but account for about 20% of deaths from COVID-19. The death rate for Blacks and Hispanics is about twice that for Whites. People who stand on one side of the political spectrum have been dying at three times the rate of those on the other side, principally because of ignorance or refusal to be vaccinated, which may be the same thing. Overall, in the U.S.A. more people have died from COVID-19 in 2021 than in 2020 and, so far, deaths due to this disease have totaled more than 130 000 in 2022. These horrible numbers may be the result of political stupidity, misinformation, or general idiocy but the death of each of the victims is a personal, familial, and economic tragedy.

Availability of vaccines seems to help. Governments are spending vast amounts of money to study the virus(es). Clinicians are hard at work to characterize and understand the disease it causes and the peculiarities of its various manifestations. Other researchers are trying to understand and identify the relationships and functions of wild vertebrates that may serve as reservoir hosts of the virus, the relationship between the size of coronavirus genomes and the mutation rate, the prevention (or allowance) of mutations in different geographic areas and among different human populations, and even more, as we recognize each day. Thus far, understanding has not led to solutions.

Many of the suggested solutions to current problems are unlikely to be carried out. National representatives and some others assigned to the United Nations have suggested nothing truly useful; they are just counting numbers. (“A committee is a cul-de-sac down which ideas are lured then quietly strangled.” – Sir Barnett Cocks)

## MONKEYPOX VIRUS DISEASE

Another recent infectious disease concern is monkeypox, a rare disease that is caused by the misnamed monkeypox virus, a member of the *Orthopoxvirus* genus in the family *Poxviridae*. The disease that monkeypox virus causes was first identified in laboratory cynomolgus monkeys in Denmark by Preben von Magnus in 1958, when two outbreaks of a smallpox-like disease occurred in colonies of monkeys captured in Malaysia, and transported via Singapore (2).

An outbreak of monkeypox virus at the Rotterdam Zoo was reported in 1964. Subsequently, monkeypox virus disease was detected in several laboratory monkeys in the U.S.A. No other cases in laboratory monkeys occurred after 1968 because conditions for housing monkeys improved and the need for monkeys, used mainly for producing the polio vaccine, from Asia and Africa decreased. The virus has not been found in Asia, and the occurrence in Asian monkeys was likely due to contracting the disease in captivity and transit, or contamination from an unknown source.

The first documented case of this disease in humans was in 1970, in an unvaccinated 9-month-old boy in Équateur Province of the Democratic Republic of the Congo (DRC; formerly Zaire). Almost 50 cases were reported between 1970 and 1979, with more than two thirds of these being from the DRC. It is likely that many more cases have occurred but not been diagnosed as such. Other cases originated from Liberia, Nigeria, Ivory Coast, and Sierra Leone. By 1986, more than 400 cases in humans had been reported. Small viral outbreaks with a death rate in the range of 10% and a secondary human-to-human infection rate of about the same percent occur routinely in equatorial Central and West Africa.

Signs and symptoms in other vertebrates vary among different species. Monkeypox-infected Gambian pouched rats (*Cricetomys gambianus*) may have mild symptoms. Some people keep these animals as pets, for reasons unknown to me. During the 2003 outbreak in the U.S.A., affected (pet!) prairie dogs (*Cynomys* species) presented with fever, cough, sore eyes, poor feeding, and rash. Non-human primates present similarly. They may have breathing problems, facial swelling, mouth ulcers, and swollen glands. In cynomolgus monkeys, the time from exposure to symptoms was noted to be about a week. Rabbits and rodents typically present with fever, cough, runny nose, sore eyes, and swollen glands. They develop small vesicles that fill with yellow fluid and may have patches of hair loss and may develop pneumonia. The disease has also been reported in dormice (*Glis* species), tree squirrels (family Sciuridae), and rope squirrels (also family Sciuridae) (3). Other animals may be susceptible.

An ongoing outbreak of human monkeypox virus disease was confirmed in May 2022, beginning with a cluster of cases found in the United Kingdom. The first recognized case was confirmed on May 6, 2022 in an individual with travel links to Nigeria, but it has been suggested that cases were already spreading in Europe in the previous months. At the time of this writing (August 31, 2022), well more than 35000 human infections with this virus have been recognized globally in the current outbreak, although numbers of case reports appear to be declining. Transmission of this virus between humans has been attributed to close contacts, perhaps even very close contact; however, this virus has been shown to be aerosol-transmitted between humans. Fewer human personal interactions likely would decrease the transmission rate, as would vaccination. There is one virus-specific vaccine approved for prevention of monkeypox, as is the vaccinia virus vaccine used to eradicate the closely related, but much more pathogenic, smallpox virus (variola virus). It is unlikely that monkeypox virus will be responsible for a pandemic, although an increasing number of cases might very well be found world-wide. Still, until the spread of this virus is stopped and awareness of it enlarged, it will not be stopped.

## CLIMATE CHANGE

Whether God or another unknown source has provided us with the abundance of resources and natural beauty, we have not taken care of our environment, as obviously is required for maintenance and, ultimately, survival of life. No need to discuss this aspect of our slovenly attempts – everyone knows what they are, even those who have become wealthy by closing their eyes to the conditions under which their children and grandchildren will live – or die.

The immediate solution to this problem is to change our energy sources from fossil fuels to highly available renewable resources. Immediately. Full stop. This will not be done, of course. When we had the opportunity to do this, we did not do it. Now we are far behind where we need to be. The causes of this disastrous hesitancy are obvious.

## UKRAINE-RUSSIA CONFLICT

Russian armed forces most recently began attacking Ukraine again on February 24, 2022. Russia is suffering tremendous losses of their fighting forces and their military equipment. Sooner or later, peace will be declared but that will require concessions, which are unlikely. A recent rumor is that Russia has offered to generously accept one-third of Ukraine territory in return for its withdrawal of Russian troops and equipment. If true, and it probably is not, this may be a reasonable request. In response to this proposal, it seems only fair that Ukraine would receive one-third of Russian territory in return.

Having all that territory in a downsized Russia would allow some breathing room (дихання номер) for the Ukrainians. They could expand their human population from about 44 million to, say, 80 million. That would still leave Russia with 30 times as much land as Ukraine. As for language differences, both speak Slavonic languages, but there is an asymmetry between them such that Ukrainians who do not speak Russian fluently can understand Russian much better than Russians, who don't speak Ukrainian fluently, can understand Ukrainian. Does not much matter now, as most Ukrainians do not want to speak with Russians anyway.

As for the patently false assertion/propaganda that Russia has attacked Ukraine to remove Nazis from power there, there probably are only four or five Nazis in Ukraine and they are all in their 90s, eating breakfast cereal three times a day in nursing homes. The former comedian-actor-lawyer Volodymyr Zelenskyy, now Ukraine’s President, has been a strong and heroic leader against Russia’s genocidal attacks. He is Jewish and part of his family was killed by Nazis during the Holocaust so, if he is a Nazi, he is not a very competent one. As for Russia, they will now have to keep working to reform their military for the next 100 years, which should keep them busy and out of trouble. In sum, as there is no valid reason for this war, the simplest solution is to simply stop fighting. Stopping doing stupid things should be easy to do.

## OTHER PROBLEMS

**Middle East conflicts:** These seem to continue *ad nauseum*. Except for the way they dress, due to my own ignorance I cannot seem to tell the difference between them. Both sides should be given one, and only one, more opportunity to make peace. Will Rogers said, “Diplomacy is the art of saying ‘Nice doggie!’ until you can find a rock.” The two sides have had more than enough time to negotiate a settlement, if both sides were serious about finding a solution to the interminable hostility between them. Enough is enough, no matter who is right and who is wrong.

Inevitably, they will not agree to peace because they see each other with their clothes on. All we (the rest of the world) need to do is make them take off their clothes in the summer and move them all to the states of North and South Dakota in North America; one group to North Dakota, the other to South Dakota; it does not matter which. Residents of the New Dakotas would have to spend the next few years producing warm clothes and foods. The land of Israel and surrounding controversial territories could be turned into an International Religious Territory, a sort of museum, with visitors limited to a few each year who win a lottery. The New Dakotans would surely complain but would be allowed to fight with each other in a sort of isolation.

**North Dakotans and South Dakotans:** Current residents of the Dakotas could be moved to the mid-Saharan desert or some other isolated and woebegone location. Most of these many places were only producing nice sweaters, oil, and camels anyway and the world already has an excess of these and certainly do not need any more.

**Dictators:** We are done with Adolf Hitler, Mao Zedong, Joseph Stalin, Pol Pot, Leopold II, Kim Il-Sung, Idi Amin, and similar if not equal tyrants, but we still have Kim Jong-un, Vladimir Putin, and more than one hundred military dictatorships in charge of various governments. Once again, the rest of the world could get together and move these people and their families to, say, an uninhabited island. After all the airports and shipping docks are destroyed, so that no one can leave or enter, the dictators can rule whatever part of the island they have dominated (that is what they do) and leave the rest of us alone. Specifically, killing women and children would remain illegal, as it already is in most civilized countries, but not in these new dictatorships.

Problems recognized; some solutions proposed.
